# Cardioprotective effects of fibroblast growth factor 21 against doxorubicin-induced toxicity via the SIRT1/LKB1/AMPK pathway

**DOI:** 10.1038/cddis.2017.410

**Published:** 2017-08-24

**Authors:** Shudong Wang, Yonggang Wang, Zhiguo Zhang, Quan Liu, Junlian Gu

**Affiliations:** 1 Cardiovascular Center, The First Hospital of Jilin University, Changchun, China; 2 Department of Pathology, Qianfoshan Hospital Affiliated to Shandong University, Jinan, China

## Abstract

Doxorubicin (DOX) is a highly effective antineoplastic anthracycline drug; however, the adverse effect of the cardiotoxicity has limited its widespread application. Fibroblast growth factor 21 (FGF21), as a well-known regulator of glucose and lipid metabolism, was recently shown to exert cardioprotective effects. The aim of this study was to investigate the possible protective effects of FGF21 against DOX-induced cardiomyopathy. We preliminarily established DOX-induced cardiotoxicity models in H9c2 cells, adult mouse cardiomyocytes, and 129S1/SyImJ mice, which clearly showed cardiac dysfunction and myocardial collagen accumulation accompanying by inflammatory, oxidative stress, and apoptotic damage. Treatment with FGF21 obviously attenuated the DOX-induced cardiac dysfunction and pathological changes. Its effective anti-inflammatory activity was revealed by downregulation of inflammatory factors (tumor necrosis factor*-α* and interleukin-6) via the IKK/I*κ*B*α*/nuclear factor-*κ*B pathway. The anti-oxidative stress activity of FGF21 was achieved via reduced generation of reactive oxygen species through regulation of nuclear transcription factor erythroid 2-related factor 2 transcription. Its anti-apoptotic activity was shown by reductions in the number of TUNEL-positive cells and DNA fragments along with a decreased ratio of Bax/Bcl-2 expression. In a further mechanistic study, FGF21 enhanced sirtuin 1 (SIRT1) binding to liver kinase B1 (LKB1) and then decreased LKB1 acetylation, subsequently inducing AMP-activated protein kinase (AMPK) activation, which improved the cardiac inflammation, oxidative stress, and apoptosis. These alterations were significantly prohibited by SIRT1 RNAi. The present work demonstrates for the first time that FGF21 obviously prevented DOX-induced cardiotoxicity via the suppression of oxidative stress, inflammation, and apoptosis through the SIRT1/LKB1/AMPK signaling pathway.

With advances in cancer treatments, the numbers of cancer survivors increased quickly.^1^ However, a medical survey of 1807 cancer survivors followed for 7 years showed that 33% died of heart diseases.^2^ Despite doxorubicin (DOX) effectiveness against cancer, dose-dependent cardiotoxicity restricts its long-term application in chemotherapy, as it diminishes the quality of life of cancer patients.^3, 4^ For example, one case report described DOX-related congestive heart failure in a patient who had received a 400 mg/m^2^ cumulative dose of DOX.^5^ The onset of cardiac complications can occur during treatment with DOX or up to 10 years after cessation of DOX therapy.^6^ In fact, cardiovascular-related disease derived from the adverse effects of cancer treatments has become the leading noncancer-related cause of morbidity and mortality in long-term cancer survivors.^6^

Despite great achievements gained over the past several decades, the precise mechanisms implicated in DOX-induced cardiomyopathy remain unclear. Oxidative stress, inflammation, and apoptosis have been proposed as the mechanisms of the DOX-induced cardiotoxicity, which at least could result in cardiac remodeling and dysfunction.^7^ To date, no targeted strategies are available for preventing DOX-induced cardiotoxicity. Some chemicals and functional factors have been evaluated for their ability to moderate DOX-induced cardiotoxicity, but little success was reported.^8, 9, 10^ Currently, dexrazoxane is the only agent approved by the United States Food and Drug Administration and the European Medicines Agency for the prevention of long-term cardiotoxicity caused by DOX. Nevertheless, dexrazoxane may interfere with the anticancer activity of DOX and lead to a higher latent risk for acute myeloid leukemia in pediatric patients.^11^ Thus, a more specific drug or strategy needs to be developed to protect against DOX cardiotoxicity.

Fibroblast growth factor 21 (FGF21), as an effective metabolic factor on glucose and lipid metabolism, was predominantly found in adipose and liver tissue. However, based on our and other’s studies, FGF21 is expressed in other tissues such as the myocardium.^12, 13, 14^ Numerous studies have demonstrated that FGF21 displays anti-inflammatory and anti-oxidative stress activities,^15, 16^ and thus has a critical role in protecting against tissue injury from acute toxicity.^17, 18^ In addition, the anti-apoptotic activity of FGF21 in islet *β*-cells and endothelial cells also was reported.^19, 20^ More recently, studies demonstrated FGF21-mediated protection against cardiac ischemia and reperfusion injury^21^ and isoprenaline-induced myocardial hypertrophy.^22^ Still, the exact effect of FGF21 and the detailed underlying mechanism on the cardiac system remain largely unclear. Our previous research demonstrated that FGF21 interacts with its receptors to stimulate sirtuin 1 (SIRT1)-dependent autophagy, which prevents diabetic cardiomyopathy.^13, 14^ Nevertheless, no study was yet explored the effect of FGF21 on DOX-induced cardiac injury.

In the present study, we first examined the protective ability of FGF21 against DOX-induced cardiotoxicity. Furthermore, the mechanisms of FGF21’s cardioprotective activity were analyzed using cardiomyoblasts (H9c2 cells), adult mouse cardiomyocytes, and a wild-type mouse model (129S1/SvImJ). The results of these experiments demonstrated that the cytotoxicity of DOX to H9c2 cells, adult mouse cardiomyocytes, and the heart of 129S1/SvImJ mice can be attenuated by FGF21. Further mechanistic studies showed that FGF21 obviously prevented the DOX-induced cardiotoxicity via the suppression of oxidative stress, inflammation, and apoptosis through activating the SIRT1/liver kinase B1 (LKB1)/AMP-activated protein kinase (AMPK) signaling pathway both *in vitro* and *in vivo*. Our findings indicate that FGF21 could be considered as a therapeutic target for the clinical treatment and prevention for DOX-induced cardiac injury.

## Results

### FGF21 prevented DOX-induced cardiac remodeling and dysfunction

**Figure 1:**
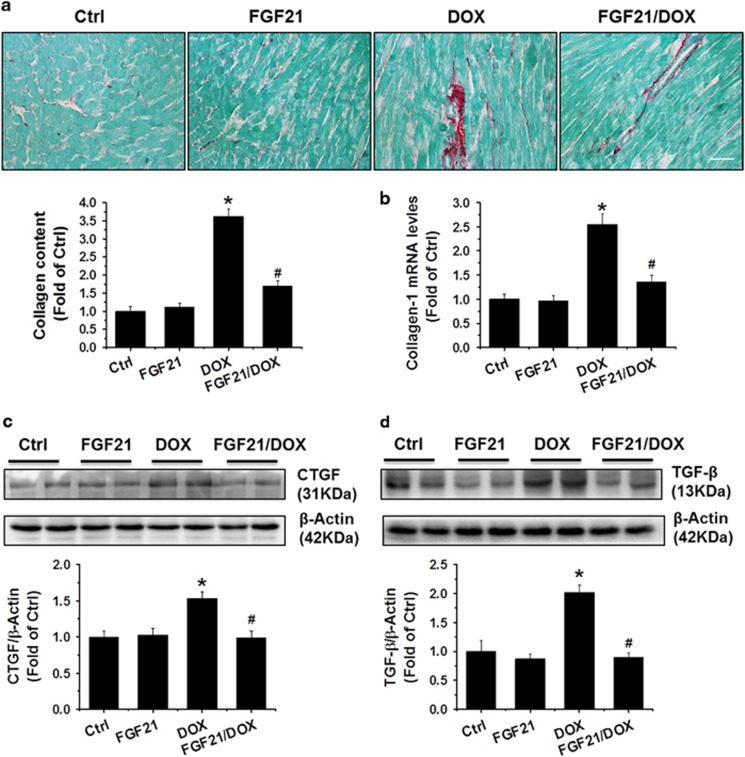
FGF21 prevented the DOX-induced cardiac remodeling and dysfunction. Sirius red staining of collagen. Scale bars=25 *μ*m (**a**), qRT-PCR of *collagen-I* (**b**), and western blotting for CTGF (**c**) and TGF-*β* (**d**) were performed to estimate the cardiac fibrotic response in heart samples of the control (Ctrl), FGF21, DOX, and FGF21+DOX treated mice. Data are presented as means±S.D. (*n*=11). *P<0.05 *versus* Ctrl group; ^#^*P*<0.05 *versus* DOX group

After administration of FGF21 to the DOX (5 mg/ml)- or PBS-treated 129S1/SvImJ mice, cardiac fibrosis was preliminary analyzed with Sirius red staining. Compared with the PBS control treatment, DOX treatment clearly caused collagen accumulation (red staining) in the cardiac slides (Figure 1a), indicating the induction of cardiac fibrosis. Pre-treatment with FGF21 significantly reduced the degree of collagen deposition induced by DOX. This result was continuously confirmed by quantitative real-time PCR (qRT-PCR) for c*ollagen I* mRNA expression (Figure 1b) and found that DOX-induced *collagen I* increase was obviously blocked by pre-administration of FGF21.

Furthermore, DOX-induced cardiac fibrosis was verified by increased expression of pro-fibrotic mediators, connective tissue growth factor (CTGF) and transforming growth factor *β* (TGF-*β*) (Figures 1c and d), and these elevations were almost completely prevented by FGF21 pre-treatment.

Next, we performed a cardiac functional analysis by echocardiography in the indicated treatment groups. DOX treatment significantly increased the left ventricular (LV) internal systolic diameter, the LV internal diastolic diameter, the LV end diastolic volume, and the LV end systolic volume, but decreased the ejection fraction (EF) and fractional shortening (FS; Table 1). Overall, pretreatment with FGF21 considerably recovered the alteration induced by DOX (Table 1).

Inflammation, oxidative stress, and apoptosis are reported to participate in DOX-induced cardiotoxicity;^23^ therefore, we subsequently investigated the protective activity of FGF21 against DOX-induced inflammation, oxidative stress, and apoptosis in the H9c2 cell line and adult mouse cardiomyocytes.

### FGF21 attenuated the upregulation of inflammatory cytokines and NF-*κ*B p65 activation in DOX-treated H9c2 cells and adult cardiomyocytes

**Figure 2:**
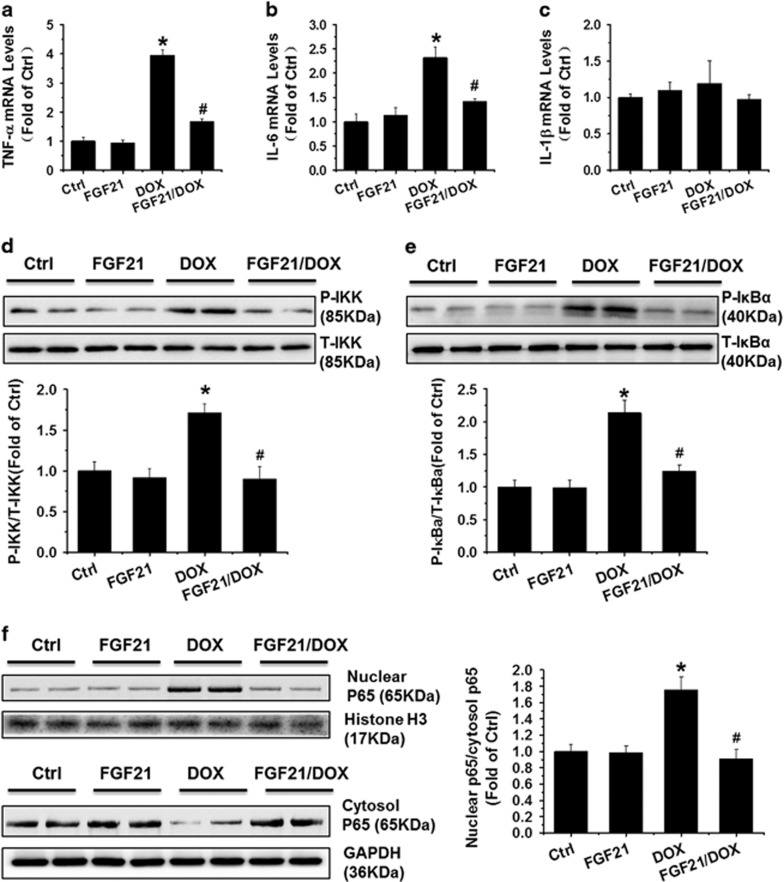
FGF21 attenuated the upregulation of inflammatory cytokines and NF-kB p65 activation induced by DOX in H9c2 cells. The mRNA levels of *TNF-α* (**a**), *IL-6* (**b**), and *IL-1β* (**c**) were examined by qRT-PCR, the phosphorylated (P-) and total proteins (T-) of the IKK (**d**) and I*κ*B*α* (**e**) were detected by western blot, and the NF-*κ*B p65 protein was detected in isolated nuclear and cytosol fractions by western blot using histone H3 and GAPDH as loading controls (**f**) in H9c2 cells treated with the indicated chemicals. Data are shown as means±S.D. of three separate experiments. *P<0.05 *versus* Ctrl group; ^#^*P*<0.05 *versus* DOX group

As shown in Figures 2a–c and Supplementary Figure 2A, DOX significantly increased the mRNA levels of tumor necrosis factor*-α* (*TNF-α*) and interleukin-6 (*IL-6*), but not *IL-1β* compared with levels in the control, which were remarkably inhibited by pretreatment with FGF21. NF-*κ*B p65 is regarded as a major controller of the transcription and expression of several inflammatory cytokines (such as TNF-*α* and IL-6),^24^ and IKK/I*κ*B*α* mediates the activation and nuclear translocation of NF-*κ*B p65. Therefore, we detected the effect of FGF21 on phosphorylated IKK (p-IKK) and I*κ*B*α* protein (p-I*κ*B*α*) expression by western blotting (Figures 2d and e). Interestingly, DOX treatment significantly increased both p-IKK and p-I*κ*B*α* expression, and these alterations were obviously attenuated by pretreatment with FGF21. Continuously, the sub-cellular distribution of NF-*κ*B p65 was analyzed by western blotting to observe whether the alteration in IKK and I*κ*B*α* affects NF-*κ*B p65 nuclear translocation. Clearly, DOX significantly increased the nuclear translocation of NF-*κ*B p65 from the cytosol and this effect was abolished by pre-treatment with FGF21 (Figure 2f and Supplementary Figure 2B).

### FGF21 efficiently prevented the DOX-induced generation of ROS and oxidative stress

**Figure 3:**
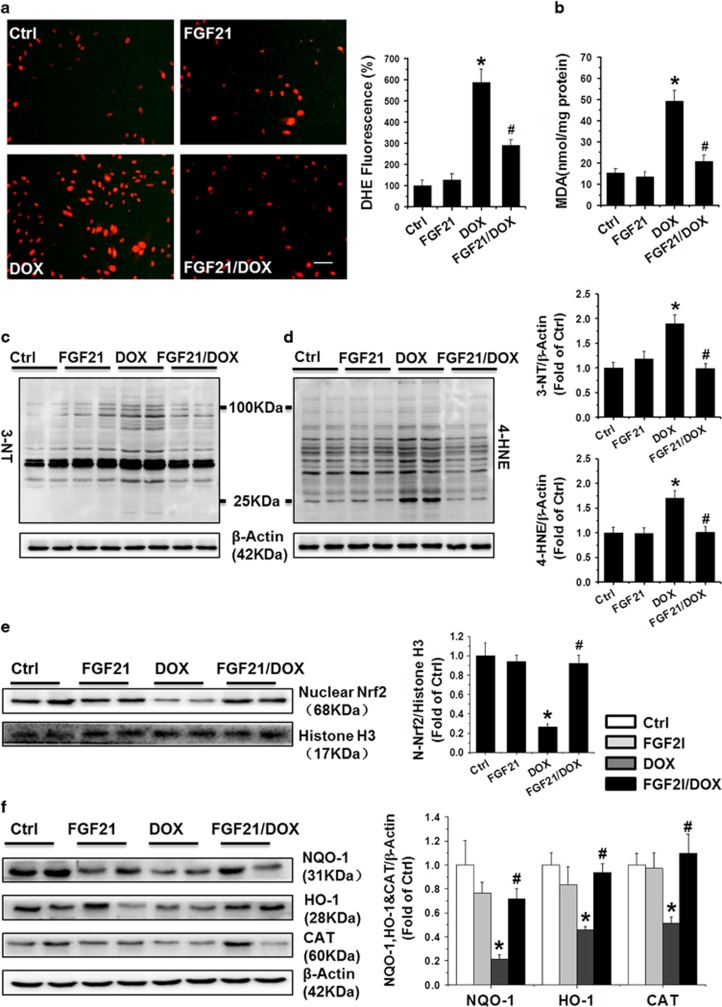
FGF21 efficiently prevented the DOX-induced generation of ROS and oxidative stress in H9c2 cells. The ROS level in the DOX-treated H9c2 cells were stained by DHE staining (Red), and the images were taken under a fluorescence microscope (scale bars, 50 *μ*m) (**a**). The lipid peroxide accumulation was quantified by malondialdehyde (MDA) assay (**b**). Accumulation of the oxidative stress markers, 3-NT (**c**) and 4-HNE (**d**), was detected by western blotting in the H9c2 cells. Accumulation of activated Nrf2 (**e**) was analyzed in the H9c2 nuclear fraction, and expression of its downstream gene products such as NQO1, HO-1, and CAT (**f**) were detected by western blotting. Data are shown as means±S.D. of three separate experiments. *P<0.05 *versus* Ctrl group; ^#^*P*<0.05 *versus* DOX group

To determine whether FGF21 can prevent the DOX-induced generation of reactive oxygen species (ROS), we measured the ROS levels in different groups. In a dihydrogen ethidium (DHE) staining study (Figure 3a), FGF21 significantly prevented the DOX-induced ROS formation in H9c2 cells. In addition, levels of the oxidative stress marker proteins 3-nitrotyrosine (3-NT) and 4-hydroxy-2-nonenal (4-HNE) (Figures 3c and d, and Supplementary Figures 3A and B), and malondialdehyde (Figure 3b and Supplementary Figure 3C) were significantly increased in the DOX group, and these increases were obviously reduced by FGF21 in the FGF21/DOX groups in both H9c2 and adult cardiomyocytes. Nuclear transcription factor erythroid 2-related factor 2 (Nrf2), as an antioxidant sensor, can translocate from the cytoplasm to the nucleus, to interact with the antioxidant defense system and mediate the transcription of target genes. According to western blot analysis (Figure 3e and Supplementary Figure 3D), nuclear Nrf2 accumulation and activity via quantification of its downstream gene such as NADPH quinone oxidoreductase-1 (NQO-1), catalase (CAT), and heme oxygenase 1 (HO-1) (Figure 3f and Supplementary Figure 3E) in the DOX group was significantly less than that in the controls, and this effect also was prevented by pre-treatment with FGF21 in both H9c2 cells and adult cardiomyocytes. Taken together, our results indicate that FGF21 attenuated DOX-induced oxidative stress, likely through restoration of a Nrf2-regulated antioxidant capacity.

### FGF21 attenuated DOX-induced cardiac apoptosis through the mitochondrial cell death pathway

**Figure 4:**
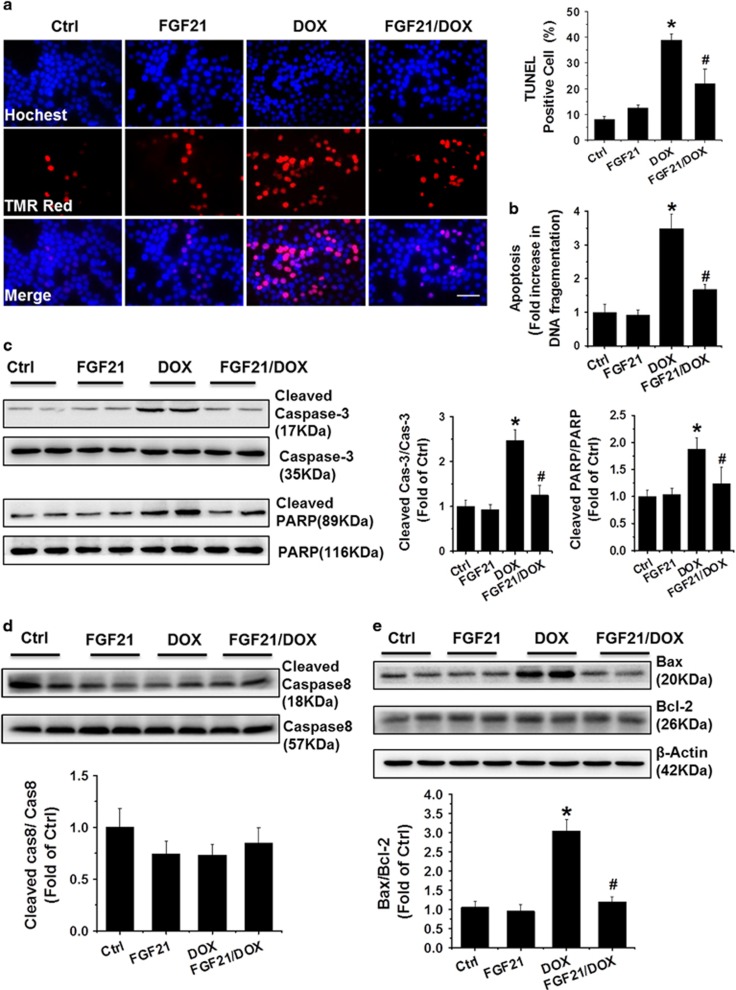
FGF21 attenuated DOX-induced cardiac apoptosis. The apoptotic H9c2 cells in different groups were detected by TUNEL staining as described (**a**) in the Materials and Methods. Scale bars=50 *μ*m. DNA fragmentation (**b**), cleaved caspase-3 (**c**), and cleaved PARP (**c**) were detected as apoptotic markers. The expression of caspase-8 (**d**) and Bax/Bcl-2 ratio (**e**) was detected by western blotting. Data are presented as means±S.D. of three separate experiments. *P<0.05 *versus* Ctrl group; ^#^*P*<0.05 *versus* DOX group

The anti-apoptotic activity of FGF21 was further examined in the DOX-induced cardiac injury model. Consistent with a previous study,^7^ DOX notably induced apoptosis in H9c2 cells and adult cardiomyocytes as detected by TUNEL staining, DNA fragmentation, and caspase-3 and (ADP-ribose) polymerase (PARP) cleavage (Figures 4a–c and Supplementary Figures 4A and B) compared with those levels in control cells. Noticeably, FGF21 completely attenuated these pro-apoptotic activities of DOX.

Accumulating evidence indicates that mitochondrial death pathways have a key role in multiple programmed cell death processes. FGF21 was reported to be involved in mitochondrial cell death pathways in diabetes-induced testicular cell death.^25^ Here we analyzed the mechanism of the anti-apoptotic activity of FGF21 in DOX-treated H9c2 cells and adult cardiomyocytes. We did not observe any significant differences in the levels of cleaved caspase-8 among the groups treated with DOX (Figure 4d). Therefore, we focused on the mitochondrial cell death pathways in our subsequent research. Western blot analysis revealed a significant increase in the Bax/Bcl-2 ratio (Figure 4e and Supplementary Figure 4C), an index of the mitochondrial cell death pathway,^26^ in the DOX group, and this change was significantly normalized by FGF21 in the FGF21/DOX group.

### FGF21 prohibited interaction of SIRT1 with LKB1 in DOX-treated H9c2 cells and adult cardiomyocytes

**Figure 5:**
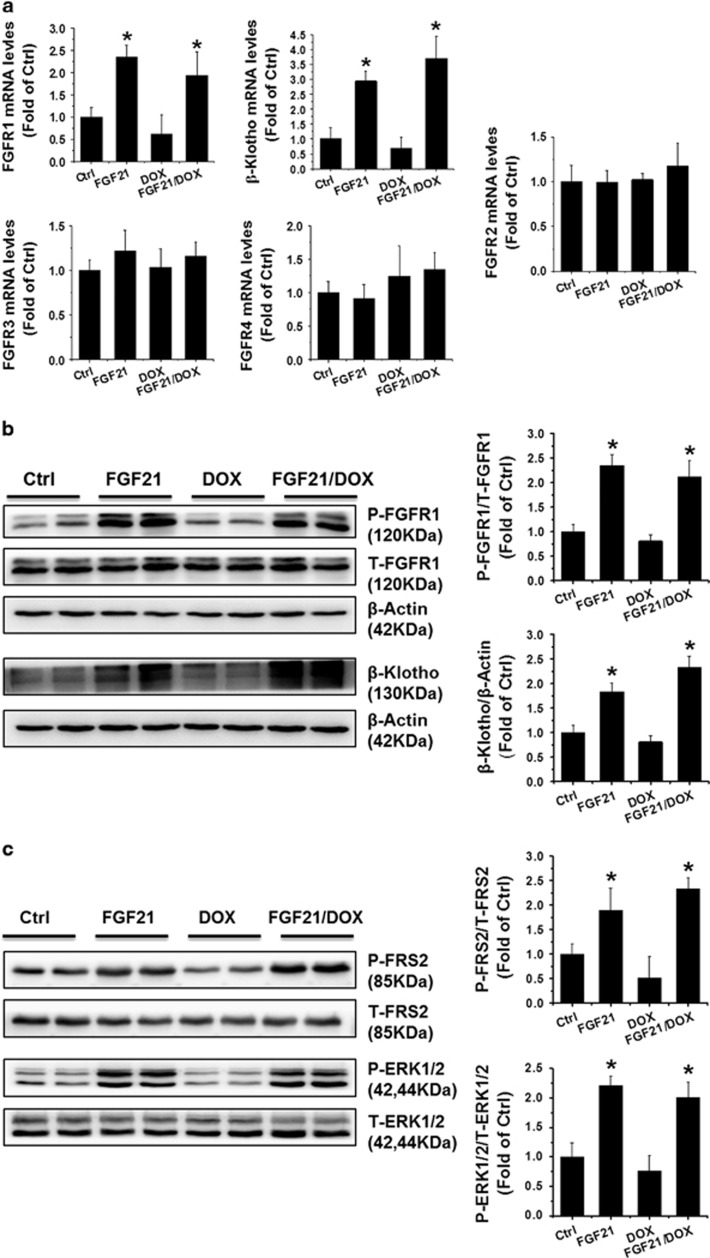

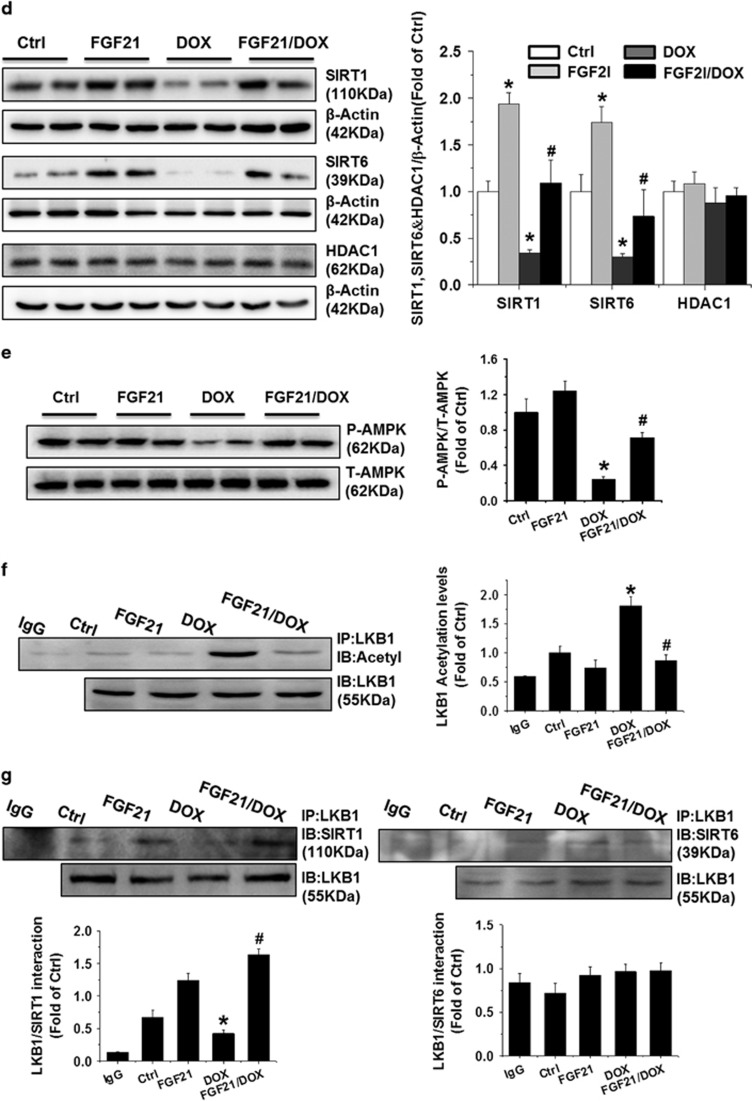
FGF21 prohibited the interaction of SIRT1 with LKB1 in DOX-treated H9c2 cells. The mRNA levels of FGFR1, *β*-Klotho, FGFR2, FGFR3, and FGFR4 (**a**) were detected by qRT-PCR. The protein expression levels of FGFR1, *β*-Klotho (**b**), SIRT1, SIRT6, HDAC1 (**d**), and AMPK (**e**) were analyzed by western blotting. The phosphorylated (P-) and total (T-) protein of FRS2 and ERK1/2 were examined by western blotting (**c**). The LKB1 proteins were immunoprecipitated with anti-LKB1 antibody in the H9c2 cells of the different groups and then probed with acetylated-lysine (Acetyl) antibodies (**f**). The LKB1 proteins were immunoprecipitated with anti-LKB1 antibody in the H9c2 cells of the different groups and then probed with SIRT1 (left panel) and SIRT6 (right panel) antibodies (**g**). Data are presented as means±S.D. of three separate experiments. *P<0.05 *versus* Ctrl group; ^#^*P*<0.05 *versus* DOX group

Our previous research demonstrated that FGF21 interacts with its receptors to prevent diabetic cardiomyopathy.^14^ Thus, the mRNA expression of both FGFR1c and *β*-Klotho receptors, which are reported as two major receptors for FGF21,^27^ as well as FGFR2, FGFR3, and FGFR4 were examined by qRT-PCR in DOX-treated H9c2 cells and adult cardiomyocytes. The results demonstrated that only FGFR1 and *β*-Klotho receptors (Figures 5a and b, and Supplementary Figure 5A), but not the other receptors, were significantly upregulated in the FGF21 and FGF21/DOX groups, and slightly decreased (but without statistical significance) in the DOX group. The phosphorylation of the signaling molecules downstream of FGF receptors were analyzed as well following FGF21 stimulation, which demonstrated that FGF21 treatment could significantly trigger the FRS2 and ERK1/2 activation in the FGF21 and FGF21/DOX groups (Figure 5c and Supplementary Figure 5B).

To investigate whether FGF21 upregulates deacetylase activity in the DOX-induced cardiac injury, we detected expression of the major nuclear histone deacetylases (HDACs), including SIRT1, SIRT6, and HDAC1. The results showed that FGF21 significantly upregulated either SIRT1 or SIRT6 activities in the FGF21 group. Conversely, DOX obviously decreased the expression of SIRT1 and SIRT6 compared with levels in the control group and these effects could be abolished by FGF21 in the FGF21/DOX group. In addition, HDAC1 expression did not significantly change among all groups (Figure 5d and Supplementary Figure 5C).

In a signaling transduction investigation, we found that FGF21 treatment significantly increased phosphorylation of AMPK in DOX-treated H9c2 cells and adult cardiomyocytes (Figure 5e and Supplementary Figure 5D). LKB1 has been verified to be the major upstream kinase of AMPK in a majority of tissues.^28^ The deacetylation status of LKB1 directly influences its intracellular localization and AMPK-regulating activity.^29^ To determine whether FGF21 mediates SIRT1/SIRT6-regulated LKB1 deacetylation, the acetylation level of LKB1 was analyzed by immunoprecipitation (IP)/western blotting. The results shown in Figure 5f and Supplementary Figure 5E indicate that FGF21 significantly decreased DOX-induced LKB1 acetylation and subsequently increased AMPK activation in the FGF21/DOX group. To explore which HDAC(s) interacts with LKB1, the nuclear extracts of the indicated H9c2 cells and adult cardiomyocytes were subjected to IP assays with SIRT1 or SIRT6 antibody as probes in LKB1-precipitated samples. Clearly, the IP tests showed that DOX prohibited the interaction of SIRT1 with LKB1 (left panel of Figure 5g and Supplementary Figure 5F), and FGF21 could notably restore this interaction of SIRT1 and LKB1 in the FGF21/DOX group. In contrast, no SIRT6-LKB1 interaction was detected among all groups (right panel of Figure 5g).

### Anti-inflammatory, anti-oxidative, and anti-apoptotic activities of FGF21 in DOX-stimulated 129S1/SvImJ mice cardiac samples *in vivo*

**Figure 6:**
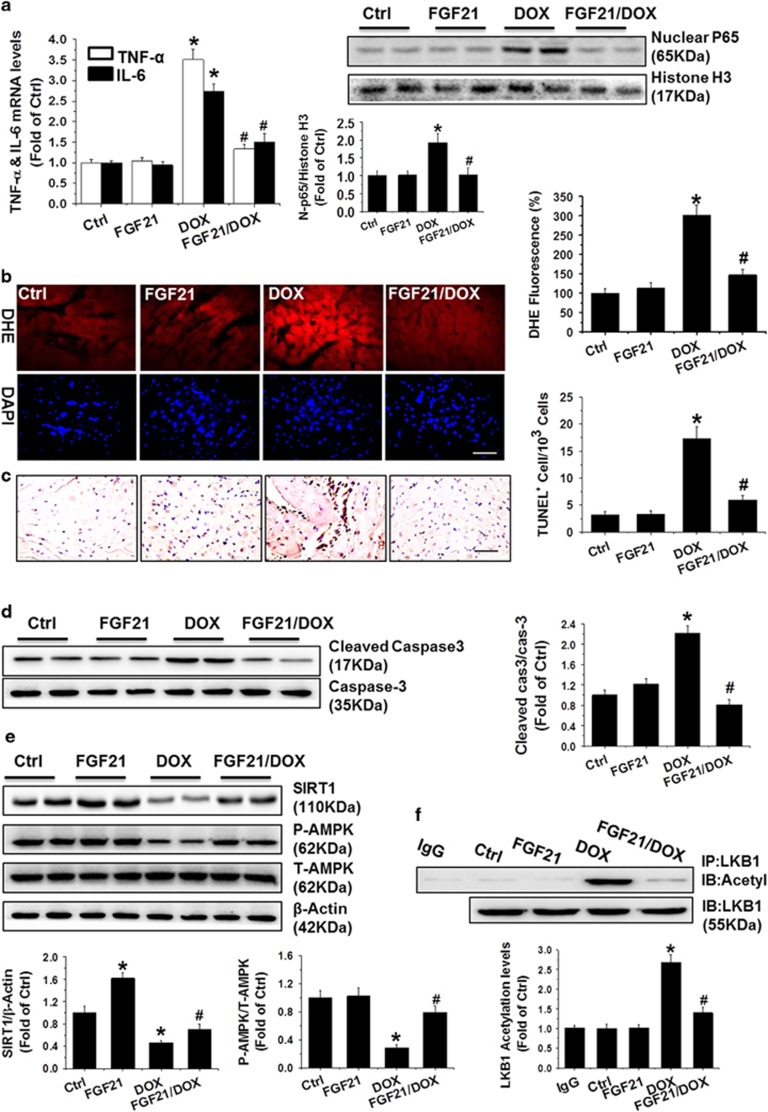
The anti-inflammatory, anti-oxidative, and anti-apoptotic activities of FGF21 in DOX-stimulated 129S1/SvImJ mice cardiac samples *in vivo*. The *TNF-α* and *IL-6* mRNA levels were analyzed by qRT-PCR in heart samples from different groups of 129S1/SvImJ mice (left panel of **a**). The NF-*κ*B p65 protein was detected in isolated nuclear fraction by western blotting, and histone H3 served as the loading control (right panel of **a**). ROS in the heart slides from different groups of 129S1/SvImJ mice were stained by DHE (Red), and images were taken under a fluorescence microscope (scale bars, 50 *μ*m) (**b**). Apoptotic cells in heart slides from the indicated groups were stained with TUNEL (scale bars=50 *μ*m) (**c**). Western blot results showed expression of cleaved caspase 3 (**d**), SIRT1 (**e**), phosphorylated AMPK (P-AMPK, in **e**), and acetylated LKB1 (detected by IP/Western blot, in F) in the heart samples from different groups. Data are presented as means±S.D. (*n*=11). *P<0.05 *versus* Ctrl group; ^#^*P*<0.05 *versus* DOX group

To confirm the above findings *in vivo* in 129S1/SvImJ mice, as shown in the left panel of Figure 6a, we found that administration of FGF21 significantly attenuated the upregulation of *TNF-α* and *IL-6* mRNA expression induced by DOX in mice by qRT-PCR. Western blot analysis revealed that the DOX-upregulated nuclear NF-*κ*B p65 protein was obviously reduced by FGF21 (right panels of Figure 6a) as well. These results verified the anti-cardiac inflammatory activity of FGF21 *in vivo*.

Continuously, the anti-oxidative stress and anti-apoptotic activities of FGF21 were detected *in vivo* using DOX-treated mice heart samples. In DHE and TUNEL staining analysis, treatment with FGF21 resulted in clear reductions in numbers of DHE-stained (Figure 6b) and TUNEL-positive (Figure 6c) cells induced by DOX. In addition, the DOX-upregulated cleaved caspase-3 (Figure 6d) was returned to normal levels after FGF21 administration, according to western blot analyses.

The signaling pathway for the ability of FGF21 to protect against DOX-induced cardiac toxicity was examined *in vivo* as well. Consistent with our previous results *in vitro*, the expression of SIRT1 and the acetylated LKB1 and phosphorylated AMPK were almost normalized by FGF21 in DOX-treated cardiac samples (Figures 6e and f).

### SIRT1 gene knockdown restricted the anti-inflammatory, anti-oxidative, and anti-apoptotic activities of FGF21 in DOX-stimulated H9c2 cells and adult cardiomyocytes

**Figure 7:**
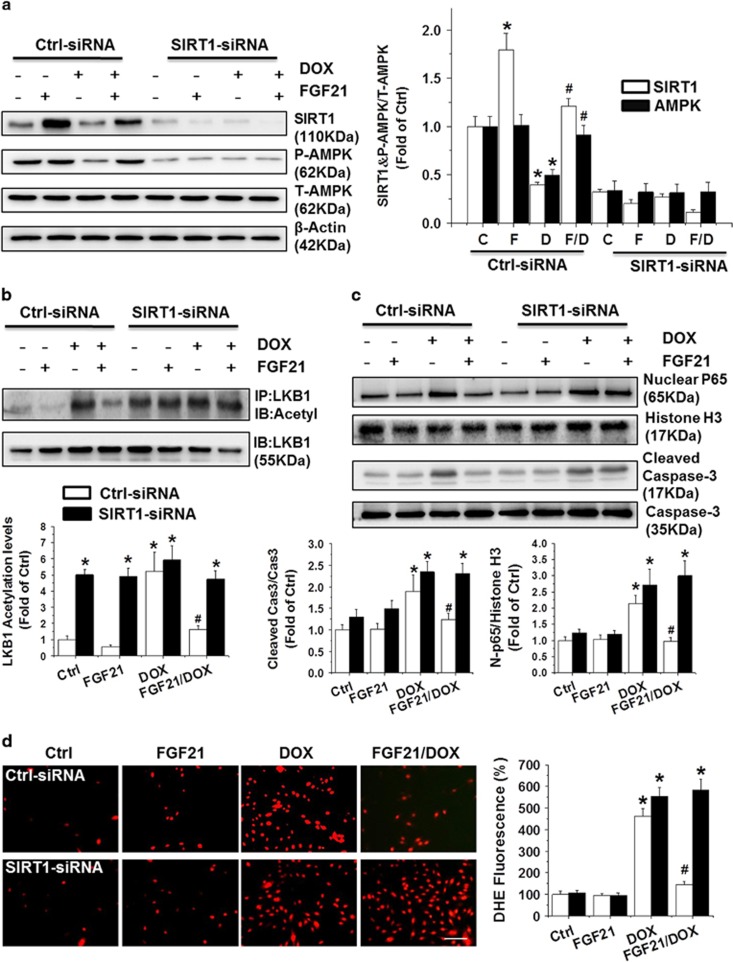
SIRT1 gene knockdown restricted the anti-inflammatory, anti-oxidative, and anti-apoptotic activities of FGF21 in DOX-stimulated H9c2 cells. The expression levels of SIRT1 and AMPK (**a**) were detected by western blotting after transfection with Ctrl-siRNA or SIRT1-siRNA in different groups. The LKB1 proteins were immunoprecipitated with anti-LKB1 antibody in the H9c2 cells of the different groups and then probed with acetylated-lysine antibodies (**b**). The protein expression levels of nuclear NF-*κ*B p65 and cleaved caspase 3 were detected by western blotting as above (**c**). The representative DHE staining (left panel) of the H9c2 cells in different groups and the quantification analysis (right panel) are presented in (**d**) (scale bars=50 μm). Data are presented as means±S.D. of three separate experiments. *P<0.05 *versus* Ctrl group; ^#^*P*<0.05 *versus* DOX group. C: Ctrl; F: FGF21; D: DOX; F/D: FGF21/DOX

To verify the critical role of SIRT1 in the deacetylation of LKB1 by FGF21, SIRT1 RNAi was employed and efficiently knocked down SIRT1 expression in both H9c2 cells and adult cardiomyocytes (Figure 7a and Supplementary Figure 6A). Interestingly, SIRT1 knockdown by RNAi almost completely recovered the level of acetylated LKB1 (Figure 7b and Supplementary Figure 6B) and subsequently prohibited the AMPK activity induced by FGF21 (Figure 7a and Supplementary Figure 6A), through which the critical role of SIRT1 in FGF21-induced activation of LKB1 and AMPK in DOX-treated H9c2 cells and adult cardiomyocytes was emphasized.

Based on this finding, the anti-inflammatory, anti-oxidative, and anti-apoptotic activities of FGF21 continued to be investigated in the SIRT1 gene knockdown H9c2 cells and adult cardiomyocytes treated with DOX. As expected, knocking down of SIRT1 by RNAi restricted the anti-inflammatory, anti-oxidative, and anti-apoptotic activities of FGF21. Compared with the siRNA control data, the capacity of FGF21 to attenuate DOX-induced inflammatory marker protein (NF-*κ*B p65, in Figure 7c and Supplementary Figure 6C), apoptotic representative protein (Caspase 3, in Figure 7c and Supplementary Figure 6C), and ROS (Figure 7d) was significantly restricted after transfection of H9c2 cells and adult cardiomyocytes with SIRT1-specific siRNA.

## Discussion

This study for the first time revealed that FGF21 can improve cardiac dysfunction and pathological changes induced by DOX. The major finding from both *in vitro* and *in vivo* experiments was that FGF21 exerts anti-inflammatory, anti-oxidative stress, and anti-apoptotic effects to attenuate the cardiac injury induced by DOX via activation of the SIRT1/LKB1/AMPK pathway.

The pathogenesis of DOX-induced cardiotoxicity involves complex processes, of which the underlying mechanisms are involved in the activation of various downstream pro-inflammatory, pro-oxidative, and pro-apoptotic processes.^30^ The increasing numbers of basic and clinical studies suggest that pro-inflammatory factors, including TNF-*α* and IL-6, participate in the pathogenesis of DOX-induced heart dysfunction.^31^ In response to the extracellular stimulation, IKK is converted from a neutral form to the active form and subsequently phosphorylates I*κ*B*α* to result in its degradation. Upon I*κ*B*α* degradation, NF-*κ*B is then released from association with the I*κ*B*α* to translocate into the nucleus where it triggers inflammatory cytokine synthesis.^32^ In our DOX-induced cardiac injury model, we preliminary found that DOX clearly elevated *TNF-α* and *IL-6* expression along with phosphorylation of both IKK and I*κ*B*α*. Pre-treatment with FGF21 not only obviously attenuated the DOX-induced upregulation of *TNF-α* and *IL-6* levels, but also decreased the levels of phosphorylated IKK and I*κ*B*α*. FGF21, in addition, significantly blocked the DOX-induced nuclear translocation of NF-*κ*B p65 (Figures 2 and 6, and Supplementary Figure 2). Taken together, these results indicate that FGF21 plays an anti-inflammatory role in the DOX-induced cardiotoxicity, and the mechanism may involve activation of IKK by FGF21 to preserve the I*κ*B*α*/NF-*κ*B p65 association. The association of I*κ*B*α*/NF-*κ*B p65 then limits NF-*κ*B p65 translocation into nuclei, thereby inhibiting transcription of inflammatory factors (such as *TNF-α* and *IL-6*).

Hearts of cancer patients receiving DOX therapy are highly sensitive to DOX-induced oxidative stress. The heart expresses low levels of antioxidant enzymes, rendering it particularly vulnerable to free radical damage and DOX cardiotoxicity.^33, 34^ In our model, multiple lines of evidence showed that DOX induced ROS generation and oxidative stress in cardiac tissue, and FGF21 exhibited the capacity to alleviate the degree of oxidative stress induced by DOX. It has been suggested that the occurrence of oxidative injury reflects the generation of ROS, which exceeds the capacity of antioxidant defense systems.^35^ Then the expression of antioxidant proteins Nrf2, NQO-1, HO-1, and CAT was found to be decreased in the DOX group, and these reductions were all significantly attenuated by FGF21. These data suggest that FGF21 possesses the free radical scavenging and antioxidant capacity via activation of Nrf2 (Figures 3 and 6, and Supplementary Figure 3).

The increase in myocardial cell apoptosis is another pathogenic mechanism in the DOX-induced cardiotoxicity.^36^ Some researchers believed that cardiomyocyte apoptosis could be the leading cause of cardiac dysfunction in DOX-induced cardiomyopathy.^37, 38^ To estimate the DOX-induced apoptosis, we conducted a series of assays including TUNEL staining, DNA fragmentation detection, and examination of the Bax/Bcl2 ratio in the DOX group. The results indicated that DOX did induce mitochondrial cell death, whereas FGF21 treatment could abolish these alterations (Figures 4 and 6, and Supplementary Figure 4).

One of our previous studies showed that FGF21 prevents lipid- or diabetes-induced cardiac apoptosis by activating the AMPK pathway^13^ and another study demonstrated that activating the LKB1/AMPK pathway prevents the development of cardiomyopathy in type 2 diabetic mice by improving lipid metabolism.^39^ These studies suggest that the LKB1/AMPK axis is an attractive therapeutic target, since it participates in multiple biological processes during cellular growth.^40^ Recent studies suggest that the LKB1/AMPK axis is a highly sensitive target of DOX-induced damage in the heart.^41, 42^ Notably, 2 *μ*M DOX (the peak plasma concentration dose of DOX) decreased LKB1/AMPK protein expression.^43^ The detailed mechanisms of LKB1/AMPK inhibition are still unclear; however, the mechanism may involve alteration of acetylation activity.

Reportedly, LKB1/AMPK signaling can be controlled through acetylation and de-acetylation by various acetyltransferases and HDACs, respectively.^44^ Among the several HDACs, SIRT1 is reported to have a critical role in controlling the cardiac LKB1/AMPK pathway. A study with *in vitro* and *in vivo* models has demonstrated the crucial role of SIRT1 for the control of inflammation and apoptosis through LKB1/AMPK-dependent pathways.^45^ In cultured 293 T cells, regulation of the LKB1/AMPK pathway by SIRT1 was also reported.^29^ In the present study, we found that FGF21 promotes interaction of LKB1 with SIRT1, then diminishes lysine acetylation of LKB1 and concurrently increases its activity, and subsequently activates AMPK to prevent the DOX-induced inflammation, oxidative stress, and apoptosis in cardiac cells (Figures 5 and 6, and Supplementary Figure 5).

It is noteworthy that SIRT1 seems to have a critical role in this pathway. To verify this hypothesis, we employed an SIRT1 RNAi to specifically block this pathway to see whether the FGF21 still prevented DOX-induced cardiac cellular inflammation, oxidative stress, and apoptosis. Undoubtedly, the SIRT1 RNAi not only prohibited the FGF21-induced LKB1 and AMPK activity, but also abolished the anti-inflammatory, anti-oxidative stress, and anti-apoptotic activities of FGF21 (Figure 7 and Supplementary Figure 6). These results verified our supposition that SIRT1 is a critical factor in FGF21-induced cardiac protection.

In conclusion, this study demonstrated that FGF21 can protect against DOX-induced cardiac toxicity. The molecular mechanism responsible for FGF21’s cardioprotective activity may involve activation of the SIRT1/LKB1/AMPK signaling pathway, which alleviates DOX-induced inflammation, oxidative stress, and apoptosis to improve cardiac dysfunction. This finding may provide an effective way to protect against or reduce the cardiac adverse effects of anthracycline during anti-cancer therapy. In addition, it delivers theoretic evidence to support the development of some more efficient and safer FGF21-like medicines such as LY2405319.^46^

## Materials and methods

### Cell culture, animal care, and experimental design

The rat cardiomyoblast line H9c2 was purchased from the American Type Culture Collection (ATCC, Manassas, VA, USA). For the *in vitro* study design, the H9c2 cells were pretreated with FGF21 at 50 ng/ml for 2 h and then incubated with DOX at 5 μg/ml for 22 h. All the experimental procedures involving animals were approved by the Institutional Animal Care and Use Committee of the Jilin University, which was in accordance with Guide for the Care and Use of Laboratory Animals, Eighth Edition (Library of Congress Control Number: 2010940400, revised 2011). Eight-week-old male 129S1/SvImJ mice were divided into four groups (Control, FGF21-, DOX-, and FGF21/DOX-treated mice) with 11 mice in each group. In the FGF21/DOX-group, the mice were given an intraperitoneal injection of 100 *μ*g/kg body weight FGF21 5 days/week for 5 weeks. This regimen included 1 week of FGF21 pretreatment before DOX exposure. One week later, the mice were intraperitoneally injected with DOX (5 mg/kg) once a week for the remaining 4 weeks. PBS injection served as a control treatment, and injection of FGF21 or DOX alone was applied in the FGF21 and DOX groups, respectively.

### Primary cardiomyocyte isolation and treatment

Adult mouse cardiomyocytes were isolated and cultured as our previous description.^13^ The adult cardiomyocytes were pretreated with FGF21 at 50 ng/ml for 2 h and then incubated with DOX at 5 *μ*g/ml for 22 h. A preliminary study was performed to optimize the transfection efficiency with SIRT1 siRNA in adult mouse cardiomyocytes (Supplementary Figure 1).

### Sirius red staining

Collagen accumulation was analyzed with Sirius red staining. The sections were incubated with 0.1% Sirius-red F3BA and 0.25% Fast Green FCF and observed under a Nikon microscope. A computer-assisted image-analysis system was applied to evaluate the sections stained for Sirius red as described before.^14^ Semi-quantitative analysis was obtained by computerization of the percentage of the positive staining from six samples in each group with two sections for each sample and five images for each section.

### Echocardiography

To assess cardiac function, transthoracic echocardiography was performed using a Philips 7500 with a 15-MHz transducer (Sonos 7500, Amsterdam, The Netherlands) as described before.^47^ Transthoracic echocardiography at the parasternal long-axis and short-axis views were performed and recorded. Two-dimensional and M-mode echo were employed to detect the wall motion, the chamber dimensions, and the cardiac function. LV dimensions and wall thicknesses were estimated with parasternal short axis M-mode images. Simultaneously, EF, FS, LV mass, and LV end diastolic volume were calculated by Philips7500 software. The final data represented averaged values of 10 cardiac cycles.

### Quantitative real-time PCR

The mRNA levels of *TNF-α*, *IL-6*, *IL-1β*, *collagen-1*, *FGFR1, β-Klotho*, *FGFR2*, *FGFR3*, and *FGFR4* were quantified by qRT-PCR as described previously.^47, 48^ The primers of *TNF-α* (Mm00443285_m1), *IL-6* (Mm00446190_m1), *IL-1β* (Mm00434228_m1), *collagen-1* (Mm01302043), *FGFR1* (Mm00438930_m1), *β-Klotho* (Mm00473122_m1), *FGFR2* (Mm01269930_m1), *FGFR3* (Mm00433294_m1), *FGFR4* (Mm01341852_m1), and *β-actin* (Mm00607939) were from Applied Biosystems (Carlsbad, CA, USA). The expression levels of the target genes were normalized to that of the housekeeping gene *β*-actin.

### Western blotting

To analyze the protein expression, western blot analysis was performed as previously described.^47, 49^ The primary antibodies to Nrf2, HDAC1, SIRT1, SIRT6, and AMPK were purchased from Abcam (Cambridge, MA, USA). Anti-LKB1 was obtained from Sigma-Aldrich (Sigma, MO, USA), anti-HO-1, anti-NQO-1, anti-CAT, anti-IKK, anti-I*κ*B*α*, anti-*β*-Klotho, anti-CTGF, and anti-TGF-*β* were from Santa Cruz Biotechnology (Santa Cruz, CA, USA). Anti-p65, anti-caspase-8, anti-caspase-3, anti-PARP, anti-Bax, anti-Bcl-2, anti-FGFR1, and anti-acetylated-lysine were purchased from Cell Signaling (Danvers, MA, USA). Anti-3-NT was from Millipore (Billerica, CA, USA), and anti-4-HNE was from Alpha Diagnostic International (San Antonio, TX, USA). After three washes with Tris-buffered saline (pH 7.2) containing 0.05% Tween 20, the membrane was reacted with appropriate secondary antibodies for 1 h at room temperature. Finally, the probed proteins were stained with enhanced chemiluminescence reagent and visualized using the BIO-RAD ChemiDoc Touch Imaging System (BIO-RAD, Hercules, CA, USA).

### Preparation of nuclear extract proteins

Nuclear protein was extracted according to the manufacturer’s instructions (Thermo Fisher Scientific, Frederick, MD, USA). The heart tissue, H9c2 cells, or adult cardiomyocytes after different treatments were harvested, centrifuged, and incubated in ice-cold buffer for 10 min. The supernatant (cytoplasmic extract) was immediately transferred to a clean pre-chilled tube and stored at −80 °C. The insoluble (pellet) fraction was resuspended and vortexed for 15 s every 10 min for a total 40 min, and after centrifugation, the nuclear protein was obtained and stored at −80 °C. For western blot analysis, histone H3, and GAPDH were used as internal controls for nuclear and cytoplasmic extracts, respectively.

### DHE staining

The DHE staining method was applied to measure the generation of ROS. The pre-treated H9c2 cells (1 × 10^6^ cells/well) or heart tissue sections (5 *μ*m thick) were incubated with 5 *μ*M/ml DHE dye (Invitrogen, Grand Island, NY, USA) in Hank’s balanced salt solution buffer for 30 min at 37 °C without light. Oxidative stress was examined and captured by immunofluorescence microscopy. The DHE fluorescence intensity (red staining) was quantified using Image Pro Plus software (Media Cybernetics Inc., Bethesda, MD, USA).

### TUNEL staining

Five-micrometer-thick tissue sections were used for TUNEL staining with ApopTag Peroxidase *In Situ* Apoptosis Detection Kit (Chemicon, Temecula, CA, USA).^13^ Briefly, the slides were deparaffinized, rehydrated, and treated with proteinase K (20 mg/ml) for 15 min at room temperature. The slide was preliminarily incubated with a TUNEL reaction mixture containing terminal deoxynucleotidyl transferase and digoxigenin-11-dUTP at room temperature for 2 h. Then hematoxylin was used for counterstaining.

The H9c2 cells (1 × 10^6^ cells/well) were seeded on six-well chamber slides. After different treatments, the slides were detected with the *In Situ* Cell Death Detection Kit, TMR red (Roche, Mannheim, Germany). Hoechst 33342 was used for nuclear counterstaining. TUNEL-positive cells were imaged under a fluorescence microscope (Nikon, Tokyo, Japan), and the cell death detection ELISA kit (Roche) was used to measure histone-bound DNA fragments as described before according to the manufacturer’s instructions.^13^

### IP assay

The IP assays were performed as described previously.^39, 49^ Briefly, H9c2 cells, adult cardiomyocytes, or heart tissue were lysed in IP buffer (25 mM Tris, pH 7.6; 150 mM NaCl; 1 mM EDTA; 1% NP-40; protease and phosphatase inhibitors). Then 300 *μ*g of the lysate was immunoprecipitated overnight at 4 °C with the monoclonal antibodies LKB1 (Sigma-Aldrich) and protein G-agarose (Pierce Biotechnology Ltd., Rockford, IL, USA). The immunocomplexes were probed by western blotting with monoclonal anti-LKB1 (Sigma-Aldrich), SIRT1 (Abcam), SIRT6 (Abcam), or acetyl-lysine antibody (Cell Signaling).

### Statistical analysis

Data are presented as means±S.D. Two-way analysis of variance for comparisons was performed for the different groups, followed by *post-hoc* pairwise repetitive comparisons with Tukey’s test, using Origin 9.0 Lab data analysis and graphing software (OriginLab Co., Northampton, MA, USA). *P*<0.05 was considered statistically significant.

## Figures and Tables

**Table 1:** Protective effect of FGF21 against DOX-induced cardiac dysfunction

	***Ctrl***	***FGF21***	***DOX***	***FGF21/DOX***
IVS;d	0.62±0.03	0.61±0.06	0.61±0.02	0.62±0.05
LVID;d	3.72±0.06	3.75±0.08	3.87±0.05*	3.77±0.07^†^
LVPW;d	0.85±0.08	0.84±0.08	0.82±0.05	0.86±0.03
IVS;s	1.03±0.07	1.07±0.08	0.99±0.08	1.03±0.02
LVID;s	2.01±0.12	2.03±0.11	2.51±0.12*	2.12±0.10^†^
LVPW;s	1.28±0.10	1.32±0.09	1.28±0.08	1.31±0.11
LV Vol;d	57.90±0.98	58.87 ±1.15	62.32±1.17*	58.69±1.41^†^
LV Vol;s	13.11±1.10	13.51±0.85	21.39±1.14*	14.79±1.92^†^
%EF	77.35±2.76	77.05±2.36	65.67±2.43*	74.70±3.87^†^
% FS	45.96±1.58	45.89±2.23	35.14±2.14*	43.76±2.74^†^
LV Mass	88.10±2.52	90.25±2.37	95.01±2.66	91.41±2.96

Abbreviations: EF, ejection fraction; FS, fractional shortening; IVS, interventricular septum; LV mass, left ventricular mass; LVID;d, left ventricular internal diastolic diameter; LVID;s, left ventricular internal systolic diameter; LVPW, left ventricular posterior wall; LV vol;d, left ventricular end diastolic volume; LV vol;s, left ventricular end systolic volume

Data are presented as means±S.D. (*n*=11). *P<0.05 *versus* Ctrl group; ^†^*P*<0.05 *versus* DOX group

## References

[bib1] DeSantis CE, Lin CC, Mariotto AB, Siegel RL, Stein KD, Kramer JL et al. Cancer treatment and survivorship statistics, 2014. CA Cancer J Clin 2014; 64: 252–271. 24890451 10.3322/caac.21235

[bib2] Vejpongsa P, Yeh ET. Prevention of anthracycline-induced cardiotoxicity: challenges and opportunities. J Am Coll Cardiol 2014; 64: 938–945. 25169180 10.1016/j.jacc.2014.06.1167

[bib3] Carvalho C, Santos RX, Cardoso S, Correia S, Oliveira PJ, Santos MS et al. Doxorubicin: the good, the bad and the ugly effect. Curr Med Chem 2009; 16: 3267–3285. 19548866 10.2174/092986709788803312

[bib4] Harake D, Franco VI, Henkel JM, Miller TL, Lipshultz SE. Cardiotoxicity in childhood cancer survivors: strategies for prevention and management. Future Cardiol 2012; 8: 647–670. 22871201 10.2217/fca.12.44 PMC3870660

[bib5] Swain SM, Whaley FS, Ewer MS. Congestive heart failure in patients treated with doxorubicin: a retrospective analysis of three trials. Cancer 2003; 97: 2869–2879. 12767102 10.1002/cncr.11407

[bib6] Lipshultz SE, Franco VI, Miller TL, Colan SD, Sallan SE. Cardiovascular disease in adult survivors of childhood cancer. Annu Rev Med 2015; 66: 161–176. 25587648 10.1146/annurev-med-070213-054849 PMC5057395

[bib7] Singh P, Sharma R, McElhanon K, Allen CD, Megyesi JK, Benes H et al. Sulforaphane protects the heart from doxorubicin-induced toxicity. Free Radic Biol Med 2015; 86: 90–101. 26025579 10.1016/j.freeradbiomed.2015.05.028 PMC4554811

[bib8] Sterba M, Popelova O, Vavrova A, Jirkovsky E, Kovarikova P, Gersl V et al. Oxidative stress, redox signaling, and metal chelation in anthracycline cardiotoxicity and pharmacological cardioprotection. Antioxid Redox Signal 2013; 18: 899–929. 22794198 10.1089/ars.2012.4795 PMC3557437

[bib9] Xi L, Zhu SG, Das A, Chen Q, Durrant D, Hobbs DC et al. Dietary inorganic nitrate alleviates doxorubicin cardiotoxicity: mechanisms and implications. Nitric Oxide 2012; 26: 274–284. 22484629 10.1016/j.niox.2012.03.006 PMC3360792

[bib10] Forman HJ, Davies KJ, Ursini F. How do nutritional antioxidants really work: nucleophilic tone and para-hormesis *versus* free radical scavenging *in vivo*. Free Radic Biol Med 2014; 66: 24–35. 23747930 10.1016/j.freeradbiomed.2013.05.045 PMC3852196

[bib11] Seif AE, Walker DM, Li Y, Huang YS, Kavcic M, Torp K et al. Dexrazoxane exposure and risk of secondary acute myeloid leukemia in pediatric oncology patients. Pediatr Blood Cancer 2015; 62: 704–709. 24668949 10.1002/pbc.25043 PMC4177031

[bib12] Fon Tacer K, Bookout AL, Ding X, Kurosu H, John GB, Wang L et al. Research resource: comprehensive expression atlas of the fibroblast growth factor system in adult mouse. Mol Endocrinol 2010; 24: 2050–2064. 20667984 10.1210/me.2010-0142 PMC2954642

[bib13] Zhang C, Huang Z, Gu J, Yan X, Lu X, Zhou S et al. Fibroblast growth factor 21 protects the heart from apoptosis in a diabetic mouse model via extracellular signal-regulated kinase 1/2-dependent signalling pathway. Diabetologia 2015; 58: 1937–1948. 26040473 10.1007/s00125-015-3630-8

[bib14] Zhang J, Cheng Y, Gu J, Wang S, Zhou S, Wang Y et al. Fenofibrate increases cardiac autophagy via FGF21/SIRT1 and prevents fibrosis and inflammation in the hearts of Type 1 diabetic mice. Clin Sci (Lond) 2016; 130: 625–641. 26795437 10.1042/CS20150623

[bib15] Yu Y, Li S, Liu Y, Tian G, Yuan Q, Bai F et al. Fibroblast growth factor 21 (FGF21) ameliorates collagen-induced arthritis through modulating oxidative stress and suppressing nuclear factor-kappa B pathway. Int Immunopharmacol 2015; 25: 74–82. 25601498 10.1016/j.intimp.2015.01.005

[bib16] Yu Y, He J, Li S, Song L, Guo X, Yao W et al. Fibroblast growth factor 21 (FGF21) inhibits macrophage-mediated inflammation by activating Nrf2 and suppressing the NF-kappaB signaling pathway. Int Immunopharmacol 2016; 38: 144–152. 27276443 10.1016/j.intimp.2016.05.026

[bib17] Feingold KR, Grunfeld C, Heuer JG, Gupta A, Cramer M, Zhang T et al. FGF21 is increased by inflammatory stimuli and protects leptin-deficient ob/ob mice from the toxicity of sepsis. Endocrinology 2012; 153: 2689–2700. 22474187 10.1210/en.2011-1496 PMC3359613

[bib18] Ye D, Wang Y, Li H, Jia W, Man K, Lo CM et al. Fibroblast growth factor 21 protects against acetaminophen-induced hepatotoxicity by potentiating peroxisome proliferator-activated receptor coactivator protein-1alpha-mediated antioxidant capacity in mice. Hepatology 2014; 60: 977–989. 24590984 10.1002/hep.27060

[bib19] Wente W, Efanov AM, Brenner M, Kharitonenkov A, Koster A, Sandusky GE et al. Fibroblast growth factor-21 improves pancreatic beta-cell function and survival by activation of extracellular signal-regulated kinase 1/2 and Akt signaling pathways. Diabetes 2006; 55: 2470–2478. 16936195 10.2337/db05-1435

[bib20] Lu Y, Liu JH, Zhang LK, Du J, Zeng XJ, Hao G et al. Fibroblast growth factor 21 as a possible endogenous factor inhibits apoptosis in cardiac endothelial cells. Chin Med J (Engl) 2010; 123: 3417–3421. 22166524

[bib21] Cong WT, Ling J, Tian HS, Ling R, Wang Y, Huang BB et al. Proteomic study on the protective mechanism of fibroblast growth factor 21 to ischemia-reperfusion injury. Can J Physiol Pharmacol 2013; 91: 973–984. 24117266 10.1139/cjpp-2012-0441

[bib22] Planavila A, Redondo I, Hondares E, Vinciguerra M, Munts C, Iglesias R et al. Fibroblast growth factor 21 protects against cardiac hypertrophy in mice. Nat Commun 2013; 4: 2019. 23771152 10.1038/ncomms3019

[bib23] Ojha S, Al Taee H, Goyal S, Mahajan UB, Patil CR, Arya DS et al. Cardioprotective potentials of plant-derived small molecules against doxorubicin associated cardiotoxicity. Oxid Med Cell Longev 2016; 2016: 5724973. 27313831 10.1155/2016/5724973 PMC4893565

[bib24] Hamid T, Gu Y, Ortines RV, Bhattacharya C, Wang G, Xuan YT et al. Divergent tumor necrosis factor receptor-related remodeling responses in heart failure: role of nuclear factor-kappaB and inflammatory activation. Circulation 2009; 119: 1386–1397. 19255345 10.1161/CIRCULATIONAHA.108.802918 PMC2730645

[bib25] Jiang X, Zhang C, Xin Y, Huang Z, Tan Y, Huang Y et al. Protective effect of FGF21 on type 1 diabetes-induced testicular apoptotic cell death probably via both mitochondrial- and endoplasmic reticulum stress-dependent pathways in the mouse model. Toxicol Lett 2013; 219: 65–76. 23499715 10.1016/j.toxlet.2013.02.022

[bib26] Martinou JC, Youle RJ. Mitochondria in apoptosis: Bcl-2 family members and mitochondrial dynamics. Dev Cell 2011; 21: 92–101. 21763611 10.1016/j.devcel.2011.06.017 PMC3156409

[bib27] Liu SQ, Roberts D, Kharitonenkov A, Zhang B, Hanson SM, Li YC et al. Endocrine protection of ischemic myocardium by FGF21 from the liver and adipose tissue. Sci Rep 2013; 3: 2767. 24067542 10.1038/srep02767 PMC3783882

[bib28] Woods A, Johnstone SR, Dickerson K, Leiper FC, Fryer LG, Neumann D et al. LKB1 is the upstream kinase in the AMP-activated protein kinase cascade. Curr Biol 2003; 13: 2004–2008. 14614828 10.1016/j.cub.2003.10.031

[bib29] Lan F, Cacicedo JM, Ruderman N, Ido Y. SIRT1 modulation of the acetylation status, cytosolic localization, and activity of LKB1. Possible role in AMP-activated protein kinase activation. J Biol Chem 2008; 283: 27628–27635. 18687677 10.1074/jbc.M805711200 PMC2562073

[bib30] Sun Z, Yan B, Yu WY, Yao X, Ma X, Sheng G et al. Vitexin attenuates acute doxorubicin cardiotoxicity in rats via the suppression of oxidative stress, inflammation and apoptosis and the activation of FOXO3a. Exp Ther Med 2016; 12: 1879–1884. 27588105 10.3892/etm.2016.3518 PMC4997971

[bib31] Saini HK, Xu YJ, Zhang M, Liu PP, Kirshenbaum LA, Dhalla NS. Role of tumour necrosis factor-alpha and other cytokines in ischemia-reperfusion-induced injury in the heart. Exp Clin Cardiol 2005; 10: 213–222. 19641672 PMC2716235

[bib32] Fuentes E, Rojas A, Palomo I. NF-kappaB signaling pathway as target for antiplatelet activity. Blood Rev 2016; 30: 309–315. 27075489 10.1016/j.blre.2016.03.002

[bib33] Ghibu S, Delemasure S, Richard C, Guilland JC, Martin L, Gambert S et al. General oxidative stress during doxorubicin-induced cardiotoxicity in rats: absence of cardioprotection and low antioxidant efficiency of alpha-lipoic acid. Biochimie 2012; 94: 932–939. 21396425 10.1016/j.biochi.2011.02.015

[bib34] Sahu BD, Kumar JM, Kuncha M, Borkar RM, Srinivas R, Sistla R. Baicalein alleviates doxorubicin-induced cardiotoxicity via suppression of myocardial oxidative stress and apoptosis in mice. Life Sci 2016; 144: 8–18. 26606860 10.1016/j.lfs.2015.11.018

[bib35] Gupta RK, Patel AK, Shah N, Chaudhary AK, Jha UK, Yadav UC et al. Oxidative stress and antioxidants in disease and cancer: a review. Asian Pac J Cancer Prev 2014; 15: 4405–4409. 24969860 10.7314/apjcp.2014.15.11.4405

[bib36] Kalyanaraman B, Joseph J, Kalivendi S, Wang S, Konorev E, Kotamraju S. Doxorubicin-induced apoptosis: implications in cardiotoxicity. Mol Cell Biochem 2002; 234-235: 119–124. 12162424

[bib37] Yang Y, Zhang H, Li X, Yang T, Jiang Q. Effects of PPARalpha/PGC-1alpha on the energy metabolism remodeling and apoptosis in the doxorubicin induced mice cardiomyocytes *in vitro*. Int J Clin Exp Pathol 2015; 8: 12216–12224. 26722406 PMC4680351

[bib38] Deus CM, Zehowski C, Nordgren K, Wallace KB, Skildum A, Oliveira PJ. Stimulating basal mitochondrial respiration decreases doxorubicin apoptotic signaling in H9c2 cardiomyoblasts. Toxicology 2015; 334: 1–11. 25997894 10.1016/j.tox.2015.05.001

[bib39] Zhang Z, Wang S, Zhou S, Yan X, Wang Y, Chen J et al. Sulforaphane prevents the development of cardiomyopathy in type 2 diabetic mice probably by reversing oxidative stress-induced inhibition of LKB1/AMPK pathway. J Mol Cell Cardiol 2014; 77: 42–52. 25268649 10.1016/j.yjmcc.2014.09.022

[bib40] Sun W, Lee TS, Zhu M, Gu C, Wang Y, Zhu Y et al. Statins activate AMP-activated protein kinase *in vitro* and *in vivo*. Circulation 2006; 114: 2655–2662. 17116771 10.1161/CIRCULATIONAHA.106.630194

[bib41] Kobashigawa LC, Xu YC, Padbury JF, Tseng YT, Yano N. Metformin protects cardiomyocyte from doxorubicin induced cytotoxicity through an AMP-activated protein kinase dependent signaling pathway: an *in vitro* study. PLoS ONE 2014; 9: e104888. 25127116 10.1371/journal.pone.0104888 PMC4134245

[bib42] Konishi M, Haraguchi G, Ohigashi H, Ishihara T, Saito K, Nakano Y et al. Adiponectin protects against doxorubicin-induced cardiomyopathy by anti-apoptotic effects through AMPK up-regulation. Cardiovasc Res 2011; 89: 309–319. 20978005 10.1093/cvr/cvq335

[bib43] Tokarska-Schlattner M, Zaugg M, da Silva R, Lucchinetti E, Schaub MC, Wallimann T et al. Acute toxicity of doxorubicin on isolated perfused heart: response of kinases regulating energy supply. Am J Physiol Heart Circ Physiol 2005; 289: H37–H47. 15764680 10.1152/ajpheart.01057.2004

[bib44] Mihaylova MM, Shaw RJ. Metabolic reprogramming by class I and II histone deacetylases. Trends Endocrinol Metab 2013; 24: 48–57. 23062770 10.1016/j.tem.2012.09.003 PMC3532556

[bib45] Zheng Z, Chen H, Li J, Li T, Zheng B, Zheng Y et al. Sirtuin 1-mediated cellular metabolic memory of high glucose via the LKB1/AMPK/ROS pathway and therapeutic effects of metformin. Diabetes 2012; 61: 217–228. 22124463 10.2337/db11-0416 PMC3237662

[bib46] Kharitonenkov A, Adams AC. Inventing new medicines: The FGF21 story. Mol Metab 2014; 3: 221–229. 24749049 10.1016/j.molmet.2013.12.003 PMC3986619

[bib47] Gu J, Cheng Y, Wu H, Kong L, Wang S, Xu Z et al. Metallothionein is downstream of Nrf2 and partially mediates sulforaphane prevention of diabetic cardiomyopathy. Diabetes 2017; 66: 529–542. 27903744 10.2337/db15-1274 PMC5248986

[bib48] Wang S, Luo M, Zhang Z, Gu J, Chen J, Payne KM et al. Zinc deficiency exacerbates while zinc supplement attenuates cardiac hypertrophy in high-fat diet-induced obese mice through modulating p38 MAPK-dependent signaling. Toxicol Lett 2016; 258: 134–146. 27346292 10.1016/j.toxlet.2016.06.020

[bib49] Gu J, Wang B, Liu Y, Zhong L, Tang Y, Guo H et al. Murine double minute 2 siRNA and wild-type p53 gene therapy interact positively with zinc on prostate tumours *in vitro* and *in vivo*. Eur J Cancer 2014; 50: 1184–1194. 24447832 10.1016/j.ejca.2013.12.027

