# Supplementary material for: Cardioprotective effects of fibroblast growth factor 21 against doxorubicin-induced toxicity via the SIRT1/LKB1/AMPK pathway
Source: Cell Death Dis. 2017 Aug 24;8(8):e3018–. doi: 10.1038/cddis.2017.410 (PMC5596591; doi:10.1038/cddis.2017.410)
Supplement: Supplementary Information [file cddis2017410x1.docx]

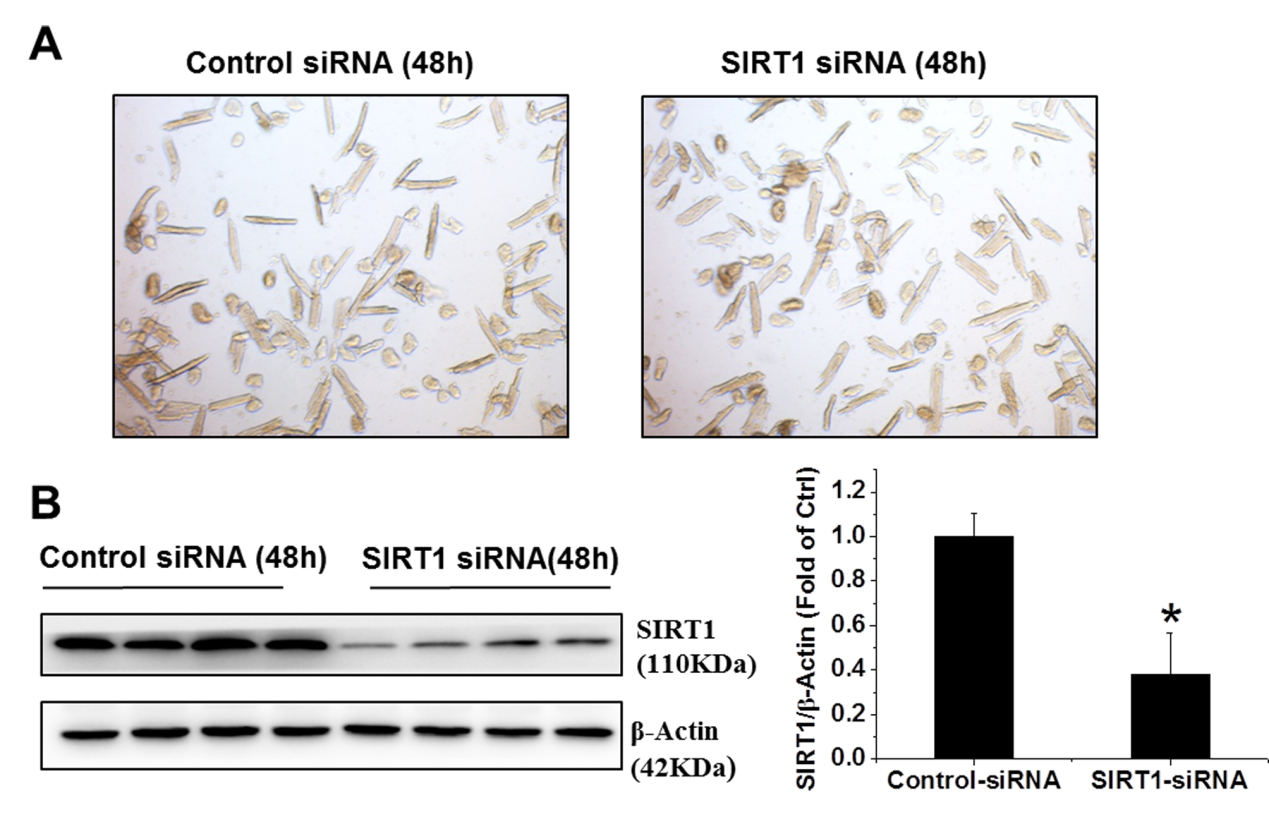


**Supplementary Fig. 1. SIRT1 gene knock-down by siRNA in primary cardiomyocytes.**

**A.** Image (20x) shows typical rod-shape primary cardiomyocytes transfected with SIRT1 siRNA (right panel) for 48 h compared with the control siRNA-transfected cells (left panel). **B**. The efficiency of the gene knock-down was assessed by western blotting (left panel). β-actin was used as the housekeeping gene reference. The expression levels of the corresponding proteins were analyzed by densitometry (right panel). Each data was calculated from four independent experiments and presented as mean ± SD. * p< 0.05 vs. control siRNA.


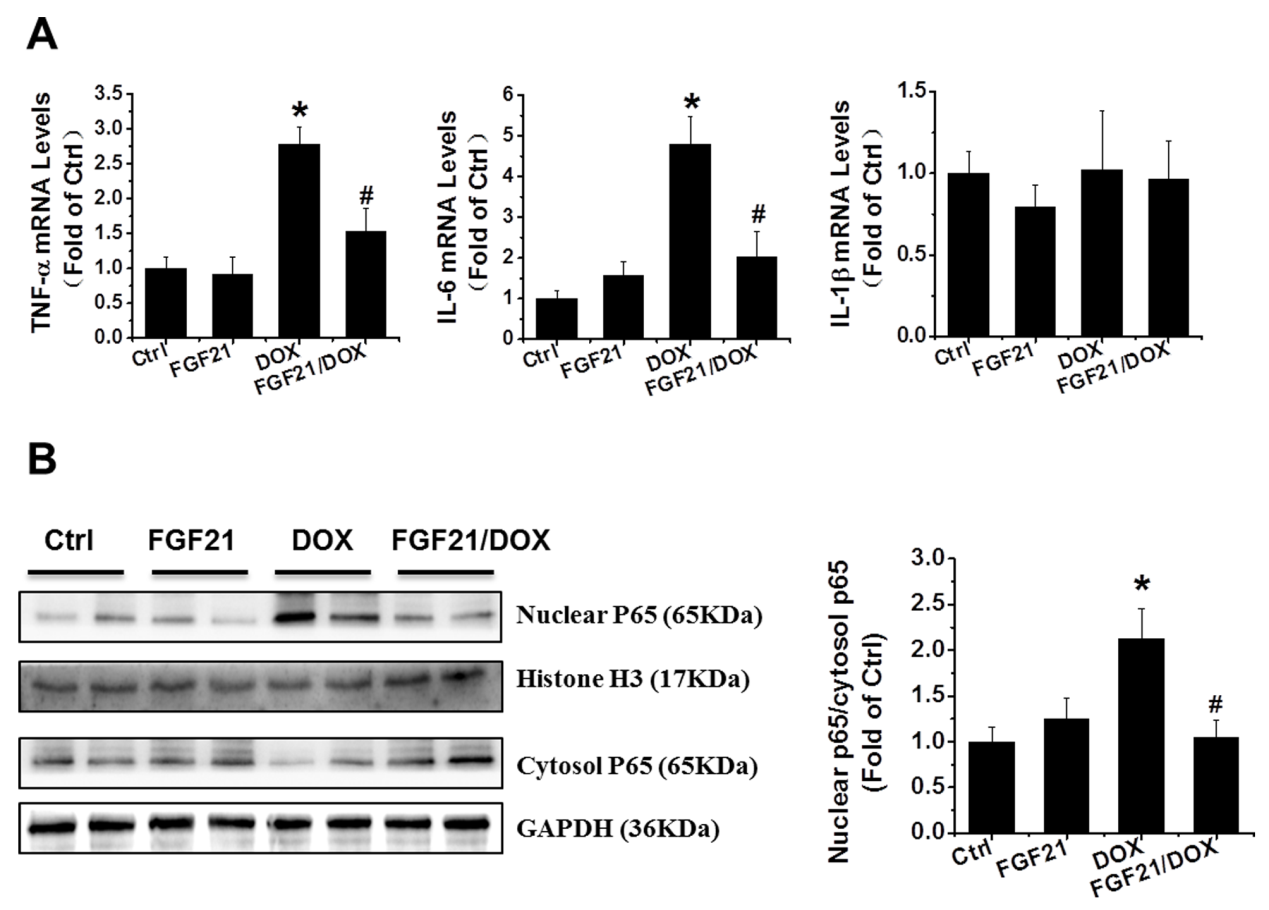


**Supplementary Fig. 2. FGF21 attenuated the upregulation of inflammatory cytokines and DOX-induced NF-κB p65 activation in adult cardiomyocytes.**

The mRNA levels of TNF-α (left panel), IL-6 (middle panel), and IL-1β (right panel) in FGF21-, DOX-, or FGF21/DOX-treated cells were examined by qRT-PCR (**A**). The NF-κB p65 protein was detected in indicated samples (FGF21, DOX, or FGF21/DOX) in isolated nuclear (1^st^ panel) and cytosol fractions (3^rd^ panel) by western blotting using histone H3 (2^nd^ panel) and GAPDH (4^th^ panel) as loading references (**B**). The densitometry of the results from B were analyzed and shown as means ± SD of three separate experiments. *, p < 0.05 versus Ctrl group; #, p < 0.05 versus DOX group.


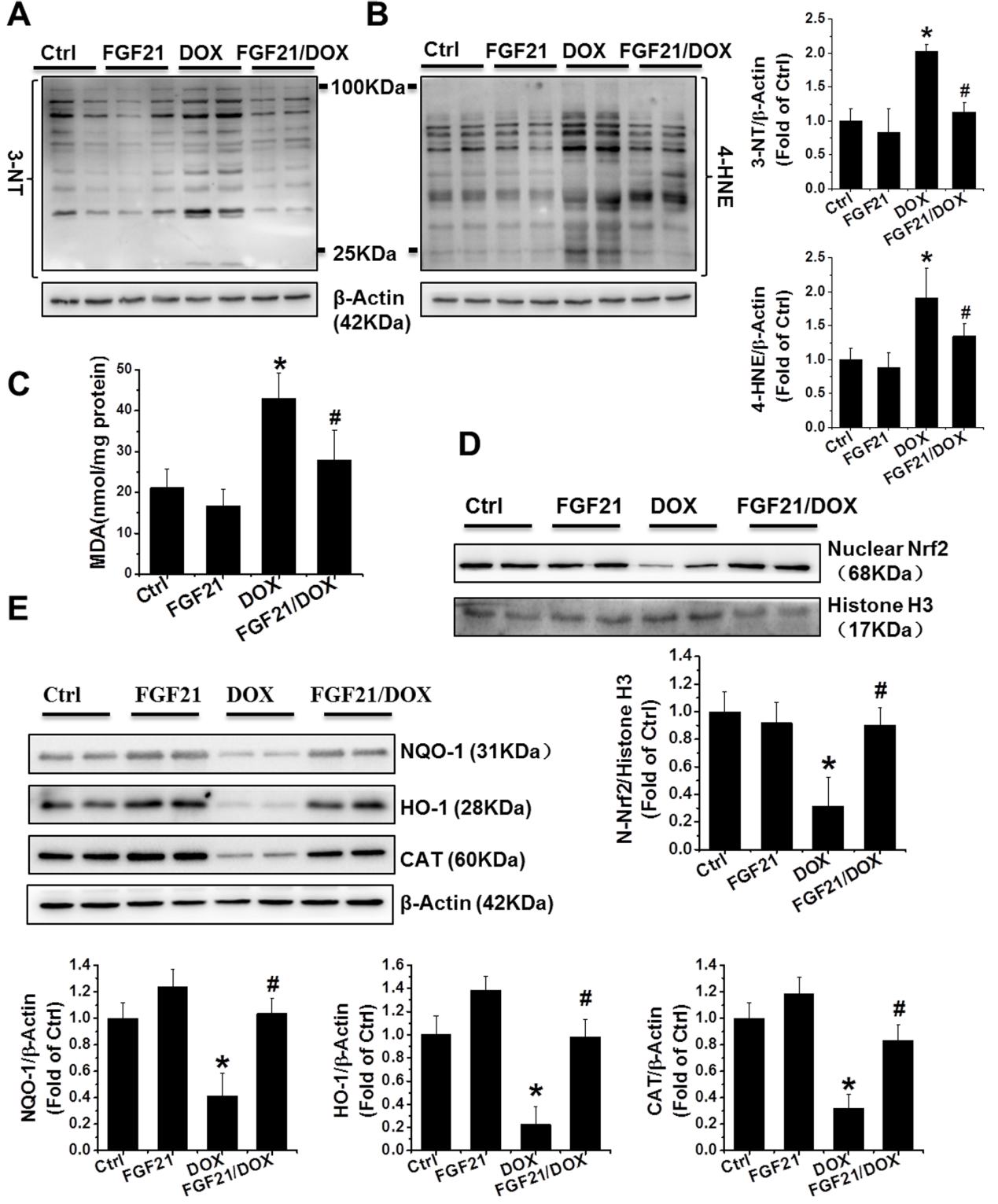


**Supplementary Fig. 3. FGF21 efficiently prevented the DOX-induced oxidative stress in adult cardiomyocytes.**

Accumulation of the oxidative stress markers, 3-NT (**A**) and 4-HNE (**B)** were detected by western blotting in adult cardiomyocytes treated with FGF21, DOX, or FGF21/DOX. The lipid peroxide accumulation of the corresponding samples was quantified by MDA assay (**C**). The accumulation of the activated Nrf2 in the corresponding groups (D) were analyzed in nuclear fraction, and its downstream genes, NQO1, HO-1, and CAT (**E**), were detected by western blotting as well. Data are shown as means ± SD of three separate experiments. *, p < 0.05 versus Ctrl group; #, p < 0.05 versus DOX group.


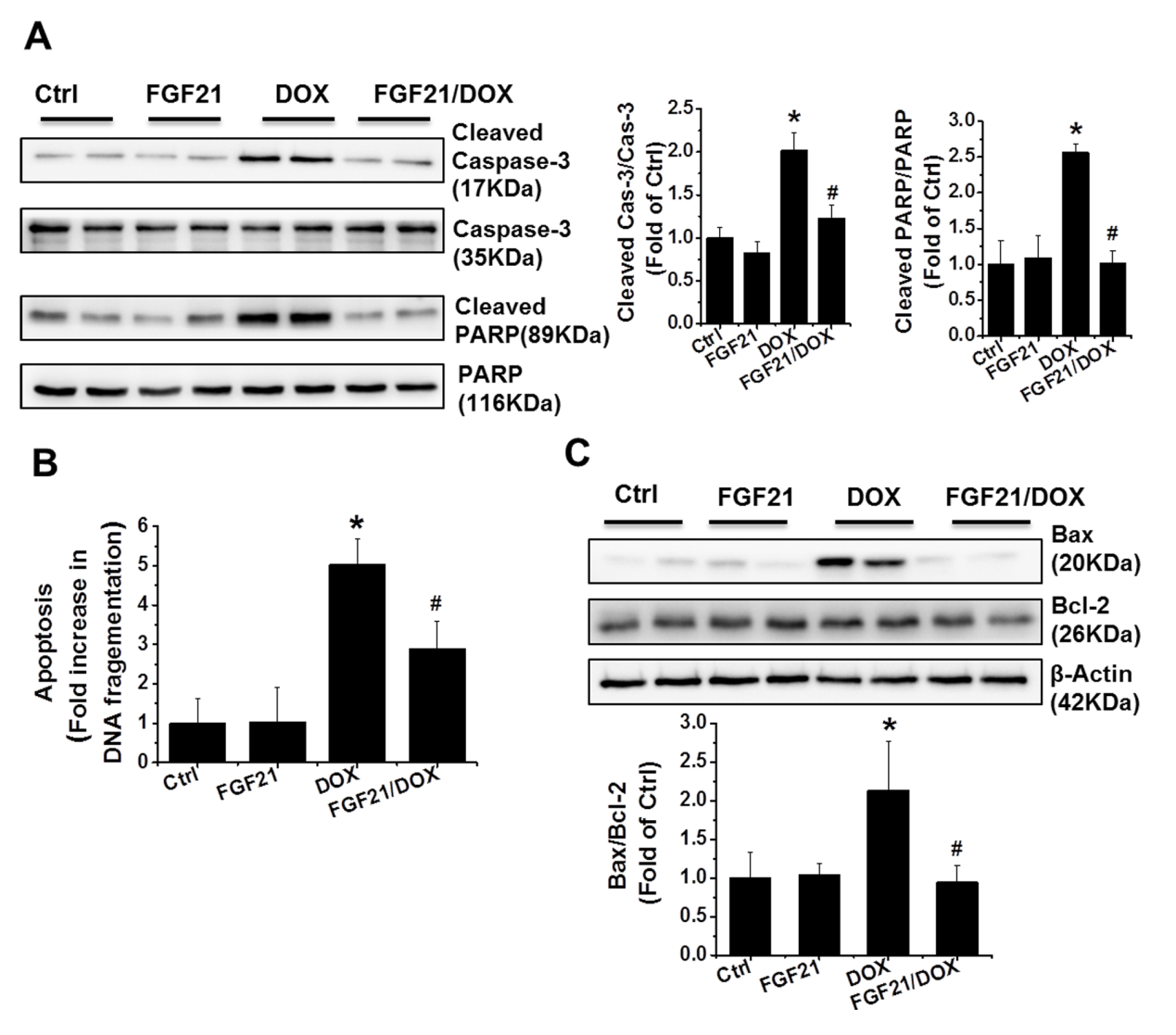


**Supplementary Fig. 4. FGF21 attenuated DOX-induced cardiac apoptosis in adult cardiomyocytes.**

The protein expression levels of cleaved caspase-3 and cleaved PARP were detected by western blotting (**A**). DNA fragmentation (an indicator of apoptotic cell death) was assessed in indicated groups by ELISA (**B**). The Bax/Bcl-2 expression ratio (lower graph) was analyzed based on the western blot assays (upper panels) (**C**). Data are presented as means ± SD of three separate experiments. *, p < 0.05 versus Ctrl group; #, p < 0.05 versus DOX group.


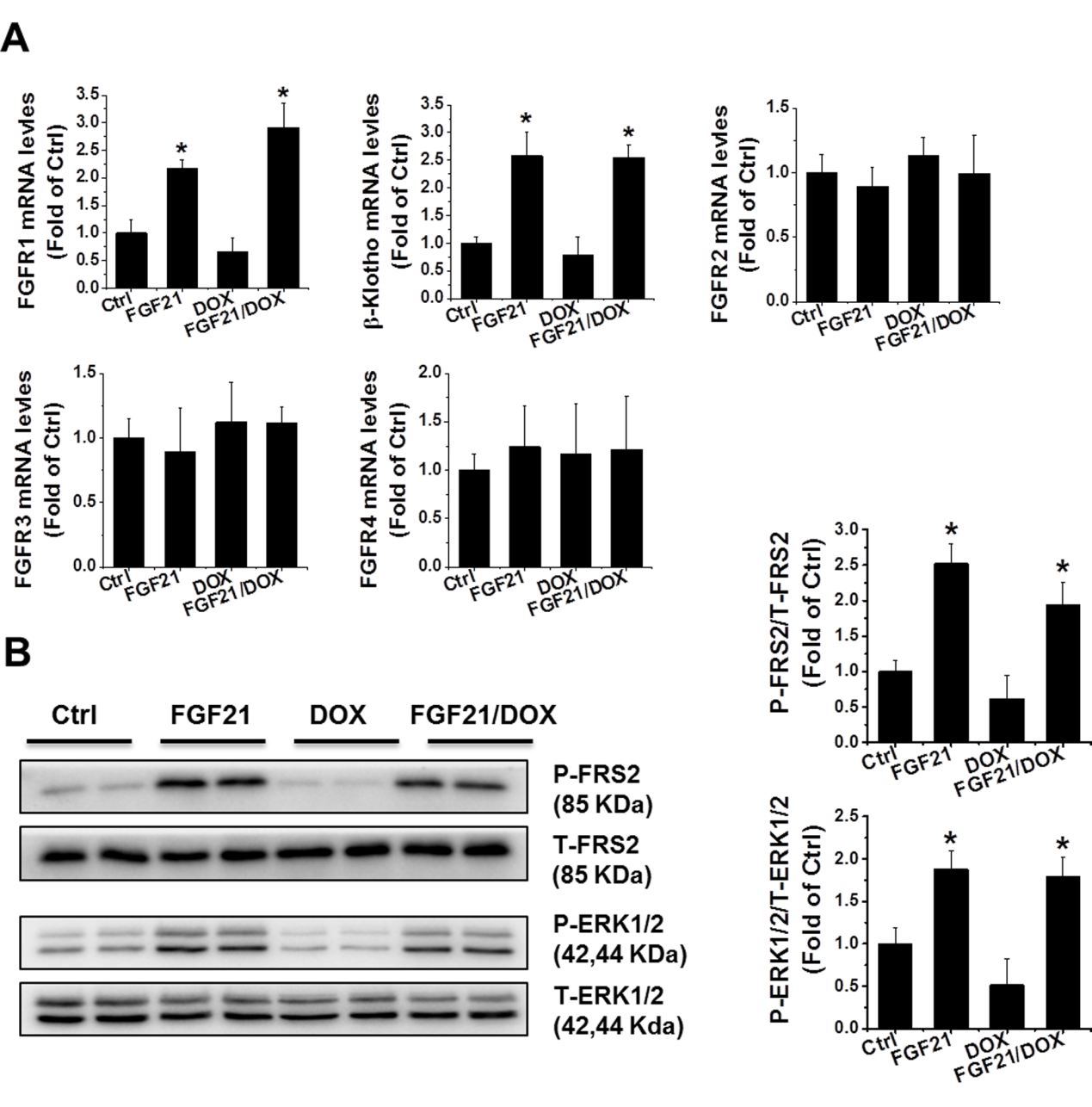


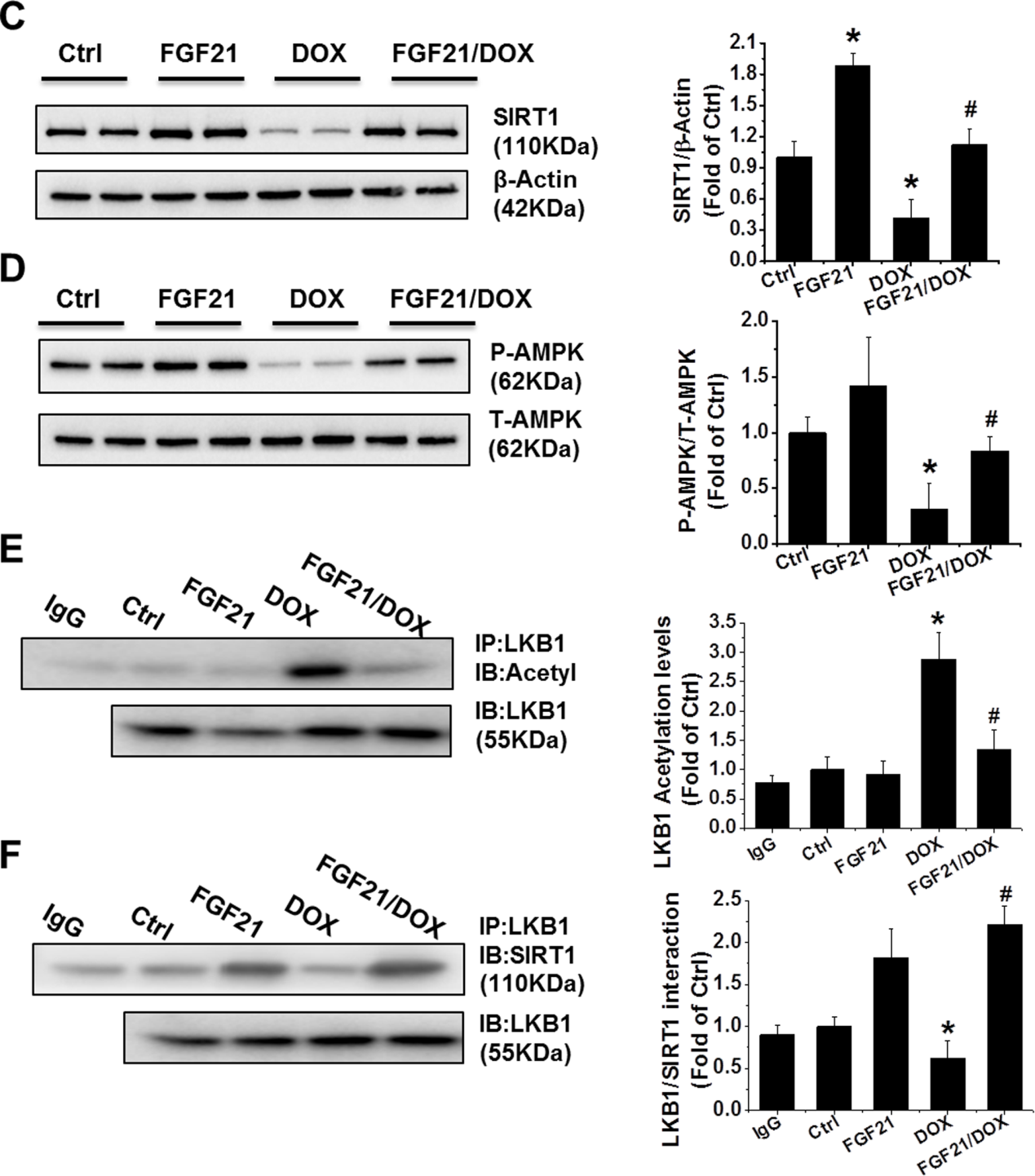


**Supplementary Fig. 5. FGF21 prohibited the interaction of SIRT1 with LKB1 in DOX-treated adult cardiomyocytes.**

The mRNA levels of FGFR1, β-Klotho, FGFR2, FGFR3, and FGFR4 were detected by qRT-PCR (**A**) in the indicated adult cardiomyocytes. The protein expression levels of FRS2, ERK1/2 (**B**), SIRT1 (**C**) and AMPK (**D**) were analyzed (right graphs) based on the western blots (left panels). The LKB1 proteins were immunoprecipitated with anti-LKB1 antibody in the adult cardiomyocytes and then probed with acetylated-lysine (Acetyl) (**E**) or SIRT1 antibodies (**F**). Data are presented as means ± SD of three separate experiments. *, p < 0.05 versus Ctrl group; #, p < 0.05 versus DOX group


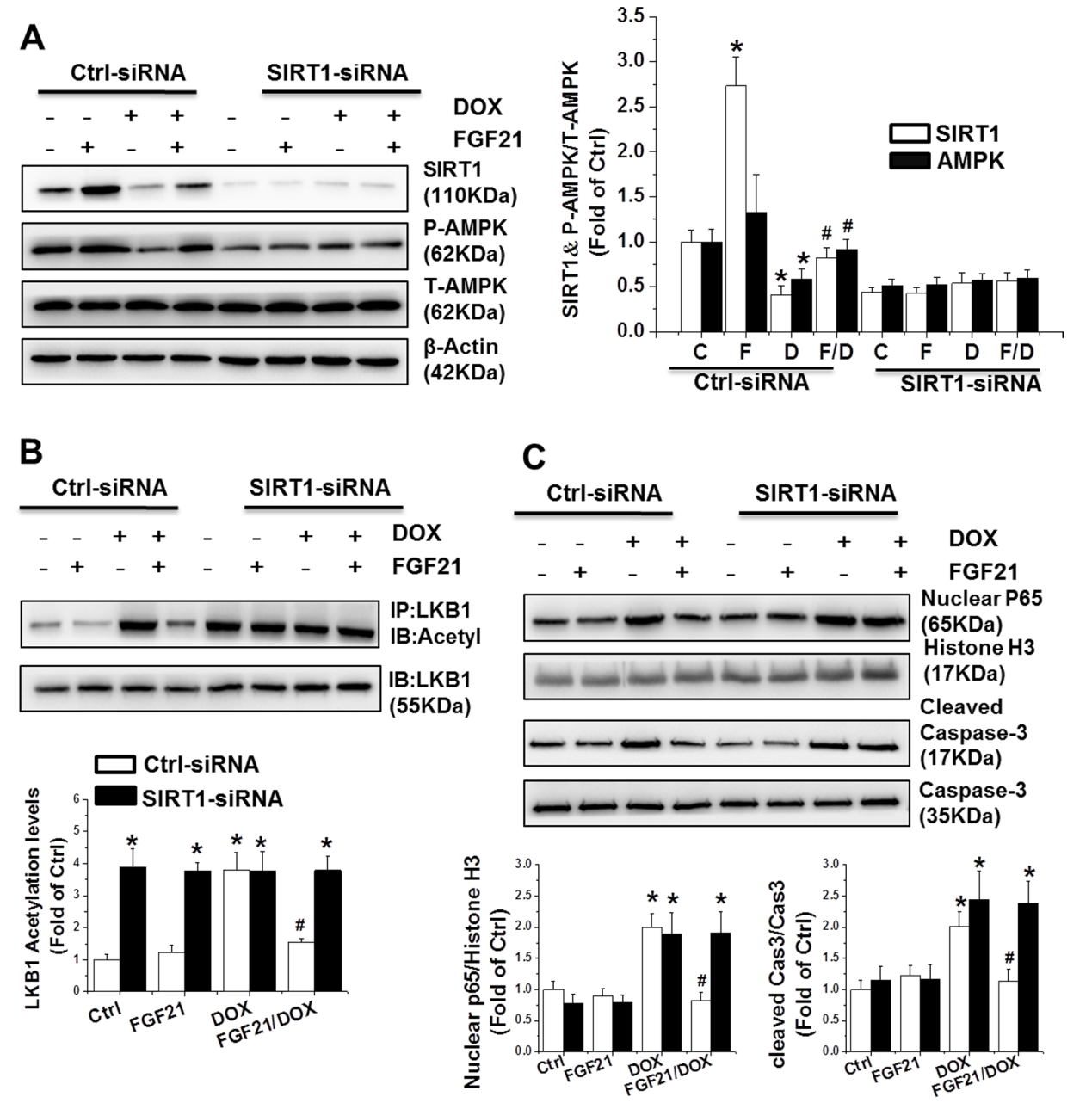


**Supplementary Fig. 6. SIRT1 gene knock-down restricted the anti-inflammatory and anti-apoptotic activities of the FGF21 in the DOX-stimulated adult cardiomyocytes.**

The expression levels of SIRT1 and AMPK (**A**) were detected by western blotting in adult cardiomyocytes transfected with Ctrl-siRNA or SIRT1-siRNA. The LKB1 proteins were immunoprecipitated with anti-LKB1 antibody in adult cardiomyocytes and then probed with acetylated-lysine antibodies (**B**). The protein expression of nuclear NF-κB p65 and cleaved caspase 3 was detected by western blotting in the same samples as above (**C**). Data are presented as means ± SD of three separate experiments. *, p < 0.05 versus Ctrl group; #, p < 0.05 versus DOX group. C: Ctrl; F: FGF21; D: DOX; F/D: FGF21/DOX.
